# Evaluation of the capability of the PCV2 genome to encode miRNAs: lack of viral miRNA expression in an experimental infection

**DOI:** 10.1186/s13567-015-0181-4

**Published:** 2015-05-01

**Authors:** Fernando Núñez-Hernández, Lester J Pérez, Gonzalo Vera, Sarai Córdoba, Joaquim Segalés, Armand Sánchez, José I Núñez

**Affiliations:** Centre de Recerca en Sanitat Animal (CReSA), UAB-IRTA, Campus de la Universitat Autònoma de Barcelona, Bellaterra, Cerdanyola del Vallès Spain; Centro Nacional de Sanidad Agropecuaria (CENSA), La Habana, Cuba; Departament de Genètica Animal, Centre de Recerca en AgriGenòmica (CRAG), CSIC-IRTA-UAB-UB, Universitat Autònoma de Barcelona, Barcelona, Bellaterra Spain; Departament de Sanitat i Anatomia Animals, Universitat Autònoma de Barcelona, Barcelona, Bellaterra Spain; Departament de Ciència Animal i dels Aliments, Universitat Autònoma de Barcelona (UAB), Barcelona, Bellaterra Spain

## Abstract

Porcine circovirus type 2 (PCV2) is a ssDNA virus causing PCV2-systemic disease (PCV2-SD), one of the most important diseases in swine. MicroRNAs (miRNAs) are a new class of small non-coding RNAs that regulate gene expression post-transcriptionally. Viral miRNAs have recently been described and the number of viral miRNAs has been increasing in the past few years. In this study, small RNA libraries were constructed from two tissues of subclinically PCV2 infected pigs to explore if PCV2 can encode viral miRNAs. The deep sequencing data revealed that PCV2 does not express miRNAs in an in vivo subclinical infection.

## Introduction, methods, and results

Porcine circovirus type 2-systemic disease (PCV2-SD) is a devastating disease that causes important economic losses [1]. The disease is essentially caused by PCV2, a single stranded DNA, non enveloped virus belonging to the C*ircoviridae* family [2]. The PCV2 genome encodes four ORFs. ORF1 encodes for two proteins (Rep and Rep’) which are involved in replication. ORF2 encodes for the Cap protein, which constitutes the unique structural protein. The two proteins coded by ORF3 and ORF4 have been related with cellular apoptosis while the function of both still needs further research [3].

MicroRNAs (miRNAs) comprise a class of small noncoding RNAs that post-transcriptionally regulate the expression of many genes by mRNA degradation or translation inhibition [4]. miRNAs are involved in the modulation of gene expression and replication of many viruses and play a pivotal role in host-virus interactions. In addition, many viruses encode miRNAs that can play a role in the infection process. To date, 308 hairpin precursors and 502 mature miRNAs have been discovered in several viruses as shown in the miRBase [5], most of them encoded by herpesviruses, which is the virus family with the highest miRNAs encoding capacity [6,7]. Other viruses belonging to the families *Polyomaviridae*, *Adenoviridae*, *Papillomaviridae, Baculoviridae* and *Ascoviridae* encode miRNAs in low numbers [8-12]. Recently, a Human Torque Teno virus, a small, ssDNA virus from the *Anelloviridae* family, that encodes a miRNA involved in interferon modulation has been described [13]. All of them share in common to be DNA viruses, with an essential nuclear phase in their replication cycle, necessary for initial miRNA biogenesis. miRNAs encoded by RNA viruses is a matter of controversy [14-16].

**Figure 1:**
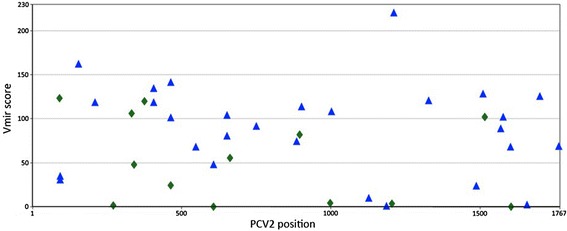
**Hairpin structures predicted for PCV2 genome of Sp-10-7-54-13 isolate (accession number GU049342) by using Vmir with default parameters.** Green diamonds and blue triangles indicate stem-loop structures in direct or reverse orientation, respectively.

In the present study, the expression of miRNAs in subclinically PCV2 infected pigs was analysed using high throughput sequencing. Firstly, in silico prediction was carried out in order to check if the PCV2 genome encodes possible miRNA precursors. The Vmir prediction algorithm [17] was used to predict the possible presence of hairpin structures in the PCV2 genome compatible with the existence of miRNAs. Computational prediction of viral miRNAs indicated that 41 miRNA candidates could be identified, 16 of them with a score among 100-150 and two with a score >150 (Figure 1). In order to explore whether these candidates were present in the virus, next-generation sequencing (NGS) of small RNAs was carried out from tonsil and mediastinal lymph node of animals subclinically infected with PCV2.

We have reported previously modification of the expression pattern of host miRNAs due to the PCV2 infection [18]. For both purposes, four animals were inoculated with 7 × 10^4.8^TCID_50_ of PCV2 isolate Sp-10-7-54-13 [19] and two animals were inoculated with PBS. Animal experiments were carried out at the CReSA facilities, all procedures were performed under the supervision of the Ethical and Animal Welfare Committee of the UAB in compliance with national guidelines and EU regulations. At 21 days post-inoculation samples were taken and total RNA extractions were carried out in order to construct small RNA libraries as described in [20] with some modifications. A total of 12 small RNA libraries were constructed in a two-step ligation procedure with the 3′ and 5′ adaptors from IDT technologies. Amplification by RT-PCR was carried out with fusion primers containing sequences complementary to the 3′ and 5′ adaptors used for miRNA library construction and sequences complementary to the A and B adaptors used for high-throughput (HT) sequencing with the GS FLX 454 device (Roche) at CRAG (Centre de Recerca Agrogenòmica, Universitat Autònoma de Barcelona, Spain). From the total reads obtained (1 106 437), primer sequences were trimmed and only those insert sequences between 15 and 29 nucleotides and with total number of sequences ≥3 were kept for further analysis. This procedure resulted in a total of 796 710 reads.

The analysis of the reads that aligned to the *Sus scrofa* genome are reported elsewhere because these sequences constitute porcine miRNAs [18]. For viral miRNA discovery, sequences were blasted to the PCV2 isolate Sp-10-7-54-13 genome (NCBI Reference Sequence: GU049342) considering only sequences with 100% of alignment and identity (perfect match). In order to search potential viral miRNAs, a blast against the viral genome was done with an increased number of mismatches in the extremes due to miRNA variability (isomiRs) [21,22]. The presence of isomiRs entails differences in length and point mutations in both extremes, with the 3′-terminus having a higher proportion of such mutations. In some cases, a blast was done allowing internal variations with a <100% of alignment in order to consider the variability of the viral genome [23]. Also, sequences were blasted to the output of Vmir hairpin PCV2 structures.

**Figure 2:**
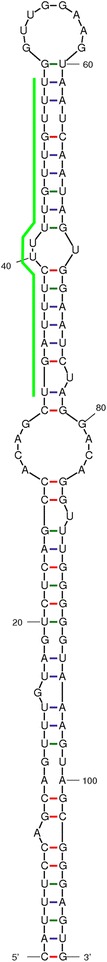
**Folding structure using MFold of pre-miRNA candidate predicted by Vmir.** The minimum free energy (ΔG) calculated was -26.60 Kcal/mol. Position of the miRNA candidate sequence detected by next-generation sequencing is indicated into the pre-miRNA structure with a green line.

**Figure 3:**
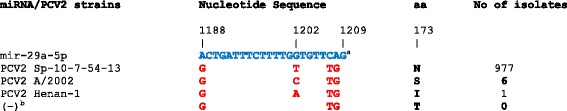
**Alignment of miR-29a-5p with positions 1188-1209 of the Sp-10-7-54-13 sequence and all PCV2 available sequences in the EMBL database.** A representative of each sequence is included in the alignment. The number of isolates with the same sequence is shown. The derived amino acid (aa position 173) from nucleotide change at position 1202 is indicated. ^a^U has been replaced by T to facilitate the understanding of the figure. ^b^No viral sequence was found in the database with this sequence.

No hits were found in the viral PCV2 genome sequence (GU049342) with a 100% homology. One candidate of 18 nt with 58 copies was identified when allowing <100% of alignment and identity in the ORF2, positions 1189 to 1206. In accordance with the Vmir prediction, these positions were included in the pre-miRNA candidate MD18, (the hairpin with the highest score), presenting one internal mismatch, and 94.4% sequence identity. The secondary structure and the minimum free energy of the pre-miRNA candidate was analysed with MFold software [24] (Figure 2). As this candidate presented a point mutation, and due to the described variability of the virus, the corresponding viral DNA fragment from the mediastinal lymph node of an infected animal was amplified and Sanger sequenced. Because the exact candidate sequence was not found in the viral sequence (94.4% sequence identity), it was not considered a miRNA encoded by the virus. The miRNA candidate sequence was compared to the porcine genome Sscrofa10.2 (GCA_000003025.4) using blast and showed 100% sequence identity with the ssc-miR-29a hairpin precursor located in chromosome 18. Also, the 18 nt miRNA candidate sequence was compared to the miRBase (v.21) showing 100% sequence identity with miR-29a-5p. The alignment of this region was carried out including all PCV2 sequences available in the databases (Figure 3). miR-29a-5p has been described in many species like human, bovine, mouse, but it has not been described in pigs, where the precursor miR-29 has been included in miRBase along with the mature ssc-miR-29a-3p. From the total reads obtained in this study, 1276 sequences of 22 nt, comprising the 18 nt of the candidate, blasted to the miR-29a-5p. From all of the above data, miR-29a-5p can be considered as a miRNA encoded by the porcine genome and the 18 nt candidate as one of the miR-29a-5p isomiRs.

## Discussion

This is the first study that tries to identify if PCV2 can encode miRNAs. PCV2 is a ssDNA virus with a nuclear phase in its replicating cycle [25]. This step is considered essential for the production of viral miRNAs, considering that the first steps in miRNA maturation take place in the cellular nucleus [26]. Nevertheless, high throughput sequencing has failed to identify any miRNA encoded by the viral genome in the natural host after an experimental infection. Thus, PCV2 may increase the list of DNA viruses not encoding miRNAs, as Cowpox virus [7]. Notwithstanding, the capacity of PCV2 to encode miRNAs has to be evaluated in cell culture, in a different clinical form or at a different time points.

Only one miRNA candidate was initially identified, but the posterior analysis indicated that it was a host miRNA, miR-29a-5p. The homology of the viral sequence with miR-29a-5p, with only one internal mismatch, led us to analyze with more detail if miR-29a-5p can regulate the expression of the Cap protein. A miR-29a-5p target prediction was evaluated using Miranda software [27] in order to explore if the Cap gen constitutes a target. As expected, a predicted target was identified in ORF2, with a low free energy of -21.65 Kcal/mol and a high complementarity score of 187. The alignment of the region with all PCV2 isolates available in the database indicated that A, C and T, but not G, has been found at position 1202 respecting Sp-10-7-54-13. All substitutions lead to a non synonymous change. The lack of the presence of G at position 1202 could be due to restriction in the protein conformation or due to the pressure exerted by miR-29a-5p in order to avoid regulation by this miRNA. If this is a consequence of viral evolution as [28] proposed, it needs further investigation. How viruses can evolve to avoid the inhibition by host miRNAs is a critical question. Some authors indicate that this evolution allows viruses to replicate without being targeted by host miRNAs by encoded viral miRNAs or modifying the expression of host miRNAs [29]. On the other hand, the secondary structure of MD18 could avoid the regulation by miR-29a-5p due to the inaccessibility of the target sequence as has been proposed for HIV [16].

In a previous study [18], in subclinically infected pigs, we demostrated that PCV2 can alter the miRNA expression pattern of the host. If PCV2 can express miRNAs in vitro has to be determined, but in its natural host, in a subclinical infection, NGS failed to indentify viral miRNAs. The exploration of the possible capacity of PCV2 to encode miRNAs could contribute to the understanding of the pathogenesis of PCV2, especially for the candidate with highest score identified by the in silico prediction. In addition, further studies on the similarity of this candidate with miR-29a-5p, and its significance, could shed light on how miRNAs affect viral evolution [30].
